# Southern Ocean biogenic blooms freezing-in Oligocene colder climates

**DOI:** 10.1038/s41467-022-34623-9

**Published:** 2022-11-09

**Authors:** Katharina Hochmuth, Joanne M. Whittaker, Isabel Sauermilch, Andreas Klocker, Karsten Gohl, Joseph H. LaCasce

**Affiliations:** 1 School of Geography, Geology and the Environment, University of Leicester, Leicester, UK; 2 Institute for Marine and Antarctic Studies, University of Tasmania, Hobart, TAS Australia; 3 Australian Center for Excellence in Antarctic Sciences, University of Tasmania, Hobart, TAS Australia; 4 Department of Earth Sciences, Faculty of Geosciences, Utrecht University, Utrecht, The Netherlands; 5 Department of Geosciences, University of Oslo, Oslo, Norway; 6 Alfred Wegener Institute Helmholtz-Centre for Polar and Marine Research, Bremerhaven, Germany; 7 Present Address: NORCE Norwegian Research Centre, Bjerknes Centre for Climate Research, Bergen, Norway

**Keywords:** Palaeoceanography, Physical oceanography, Geophysics

## Abstract

Crossing a key atmospheric CO_2_ threshold triggered a fundamental global climate reorganisation ~34 million years ago (Ma) establishing permanent Antarctic ice sheets. Curiously, a more dramatic CO_2_ decline (~800–400 ppm by the Early Oligocene(~27 Ma)), postdates initial ice sheet expansion but the mechanisms driving this later, rapid drop in atmospheric carbon during the early Oligocene remains elusive and controversial. Here we use marine seismic reflection and borehole data to reveal an unprecedented accumulation of early Oligocene strata (up to 2.2 km thick over 1500 × 500 km) with a major biogenic component in the Australian Southern Ocean. High-resolution ocean simulations demonstrate that a tectonically-driven, one-off reorganisation of ocean currents, caused a unique period where current instability coincided with high nutrient input from the Antarctic continent. This unrepeated and short-lived environment favoured extreme bioproductivity and enhanced sediment burial. The size and rapid accumulation of this sediment package potentially holds ~1.067 × 10^15^ kg of the ‘missing carbon’ sequestered during the decline from an Eocene high CO_2_-world to a mid-Oligocene medium CO_2_-world, highlighting the exceptional role of the Southern Ocean in modulating long-term climate.

## Abstract (web summary)

A phase of unique turbulent oceanographic and tectonic circumstances during the Early Oligocene caused high productivity in the Australian Antarctic Basin and enabled the stabilization of colder global climates.

## Introduction

**Fig. 1:**
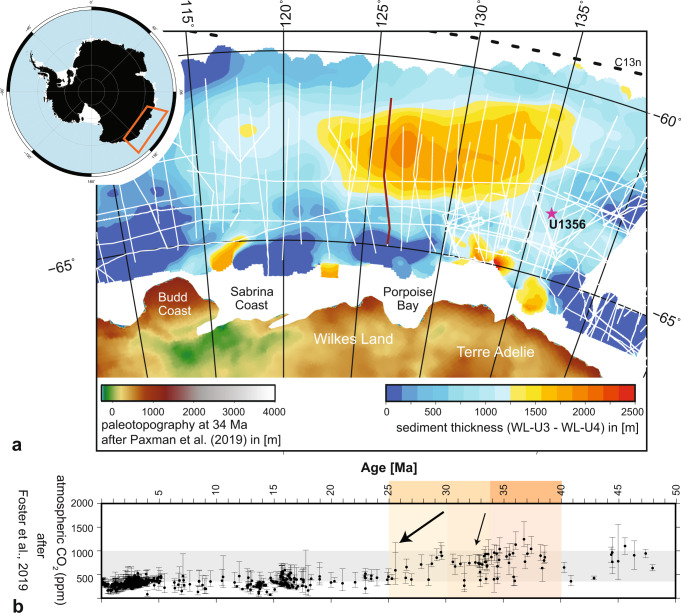
Sedimentation and atmospheric CO_2_ during the early Oligocene. **a** Sediment thickness of the Early Oligocene Strata (EOS) offshore Wilkes Land, East Antarctica. White lines indicate available seismic reflection data, magenta star indicates IODP Site U1356. Paleotopography on continent is masked to modern continental outline^31^. Black dashed lines indicate magnetic anomaly C13n (33.5 Ma)^57^, representing the approximate location of the spreading ridge in the Early Oligocene. The location of seismic profile GA2238-23 (Fig. 2) is shown in dark red. **b** Cenozoic atmospheric CO_2_ (ppm)^12^. Gray field marks CO_2_ corridor between 1000 and 400 ppm, and orange colored field relates to the studied time slices of the late Eocene and Oligocene (see Fig. 4). Black arrows indicate time frames of significant drop in atmospheric CO_2_ at the Oi-1 event (small arrow) and between 30 and 25 Ma (thicker arrow). Error bars are indicated as published by Foster et al.^12^.

Atmospheric CO_2_ levels are a key controlling factor for future and past global climate changes. During the Eocene-Oligocene, sedimentary proxy records broadly show that CO_2_ declined from >1000 parts per million (ppm) 34 million years ago (Ma) to ~400 ppm by ~27 Ma^1,2^ (Fig. 1). This decline, which includes the crossing of a proposed atmospheric CO_2_ threshold^3–5^, has been attributed to the rapid expansion of the East Antarctic ice sheet^6–10^ and the transition from a warm-house to a cold-house climate mode^11^. The threshold value itself is highly dependent on model configuration (~560–920 ppm)^4^. Analysis of various proxy datasets reveal two main stages of CO_2_ decline, the rapid overshoot decline of atmospheric CO_2_ at the Oi-1 oxygen isotope event at 33.7 Ma followed by a period of overshoot correction and a second step of CO_2_ decline at the end of the early Oligocene (Fig. 1)^1,12^. Despite the critical importance of rapidly declining CO_2_ concentrations, regardless of a threshold value, as the primary driver of global climate change, the mechanism responsible for this dramatic CO_2_ drawdown remains controversial^13,14^ and the final location of the carbon sequestration unknown.

As several global reorganization events in the ocean and atmosphere occurred simultaneously during this critical time period of the early Oligocene (34–28 Ma), different processes and biogeochemical feedback mechanisms for CO_2_ drawdown and carbon sequestration have been proposed to explain short-term and long-term mechanisms: global sea level fall enhancing carbonate weathering on newly exposed continental shelves^13,15^; dropping carbonate compensation depth^16^ and booming primary productivity in the oceans^6^. Particularly, the latter is supported by marine sediment records in the Southern^1,17^ and Equatorial^6,18^ oceans suggesting that the majority of sequestered carbon lies now in these regions^6^. Such high amount of carbon sequestered within this relatively short time period should be reflected in an abnormally high sedimentation rate and increased organic carbon content in the sediments and therefore should be detectable with geophysical methods and sedimentary drill data. Here, we show that unique oceanographic circumstances temporarily enhanced the carbon drawdown potential of the Australian–Antarctic Basin in the early Oligocene manifesting global colder climates.

## Results and discussion

### Sedimentation in the deep-sea offshore Wilkes Land

**Fig. 2:**
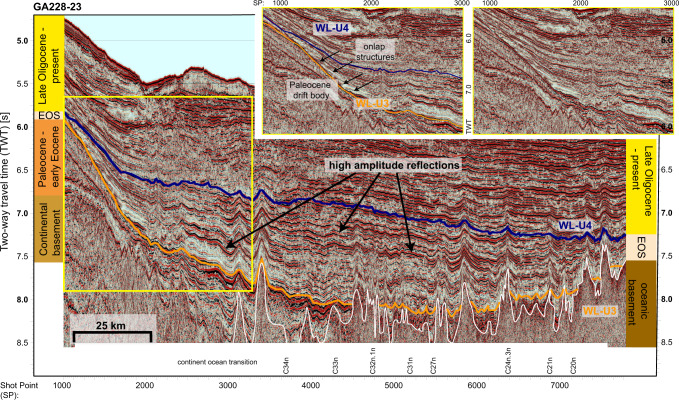
Seismic reflection profile GA228-23: interpreted seismic reflection profile GA228-23 offshore Porpoise Bay (for location see dark red line in Fig. 1). Orange line marks unconformity WL-U3 (hiatus 48–34 Ma), blue line marks unconformity WL-U4 (27 Ma). High amplitude reflectors indicate chert-like deposition. The white reflector indicates the oceanic basement. Seafloor magnetic anomaly identifications^57^ span ages from Early Cretaceous (C34n) to the early Eocene (C20n). Insets show close-up of onlap structures (yellow box in main figure) of early Oligocene strata (EOS) onto Paleocene strata. An uninterpreted version of this profile is available as Supplementary Fig. 4.

We analyzed and interpreted all available multichannel seismic reflection profiles offshore the Antarctic margin and its conjugates, which have been correlated to the Southern Ocean sediment drill sites (see Supplementary Information for methods). In the Australian–Antarctic Basin (AAB), the data reveal an abnormally thick, sedimentary package of early Oligocene deposition (Figs. 1 and 2 and Supplementary Fig. 2), which is unique to the Southern Ocean throughout the Cenozoic (see sediment thickness maps^19^). This previously unidentified Early Oligocene Strata (EOS), located on the abyssal plain, is of remarkable size (extending ~450 km latitudinally × 1500 km longitudinally) and thickness (up to 2.2 km at 127°E). No similar sediment package deposited at such a size, thickness and rate could be identified in seismic reflection data^19^ elsewhere in the abyssal plain of the Southern Ocean during the Cenozoic or in earlier times.

EOS is bounded by distinctive seismic reflection horizons WL-U3 at the base and WL-U4 at the top (Fig. 2). These horizons exhibit tremendous divergence, representing increased sediment thickness with distance from the Antarctic margin into the deep sea. Traced back to IODP Site U1356, proximal to the Antarctic coast where EOS is relatively thin, these horizons constrain deposition between 34 and 27 Ma (Supplementary Figs. 1 and 2). Horizon WL-U3 is marked by a strong consistent reflection, which is traceable across the entire basin and represents a sedimentary hiatus between 48 and 34 Ma at Site U1356. The comparison with the conjugate Australian margin infers non-deposition and strong currents as a formation mechanism^17,20,21^. Horizon WL-U4 has been dated at 27 Ma at Site U1356 and marks the onset of ocean bottom current-related re-deposition along the East Antarctic continental shelf by the flow of the Antarctic Coastal Counter Current^22^. Both horizons signify basin-wide developments and are connected to IODP site U1356 through multiple cross-sections (Fig. 1). The strata recovered for the early Oligocene at U1356 are interbedded, bioturbated claystones with silt-laminated claystones and minimal clast abundance (lithostratigraphic unit VII^23^). It is important to emphasize, that U1356 (3992 m water depth) is located on the continental rise of Wilkes Land outside of the area of extreme high sedimentation. Therefore, sedimentation mechanisms and depositional environments can only partly be related to the deep-sea environment, where EOS has formed.

By extrapolating the age of the reflectors signifying basin-wide synchronous developments at 34 and 27 Ma, measured in the very eastern part of the EOS throughout the entire basin, we calculate a mean sedimentation rate of 16 cm/kyr and a maximum of up to 30 cm/kyr around 127°E (Fig. 1). Our estimates represent a minimum as they do not account for sediment unloading and decompaction or potential small-scale erosion.

In the modern open ocean, pelagic sedimentation rates of 16–30 cm/kyr are extremely high. Comparable sedimentation rates of up to 20 cm/kyr are only known from the polar front during deglaciation^24^ and have not been observed sustained over multi-million-year timescales. Although there is strong evidence for increased productivity globally^6,16,25^ and in the Southern Ocean^17^ during the early Oligocene, most ocean basins within the Southern Ocean exhibit relatively low sedimentation rates (1–2.5 cm/kyr) as well as the onset of major ocean-current controlled re-deposition^19^.

Onshore erosional and transport processes such as large delta systems or glacial trough mouth fans typically drive rapid deposition of large sedimentary packages. For example, comparable sedimentation rates in the Southern Ocean are only reported within sediment drift systems, on continental shelves and shelf breaks, e.g., upper Pliocene glacially sourced sediment in the Ross Sea (IODP U1523, 23 cm/kyr)^26^ and other glacial outlets^19^.

However, in the case of EOS, observations do not support glacially derived terrigenous sediment as the main source, despite East Antarctic-wide glaciation in the Eocene/Oligocene transition. Evidence includes:

Observation of down-slope channel-levee and mass-transport deposition, which is very prominent at this margin in later glacial stages^27–29^ is mostly absent from the seismic data of the early Oligocene. The onset of small-scale channel-levee systems, e.g., offshore the Totten glacier has been reported for the upper Oligocene section after the deposition of the EOS strata^28^.

Close to the continental slope, EOS onlaps onto large well preserved sediment drift bodies formed during the Paleocene^21^ (Fig. 2). Significant down-slope sedimentation would either cover these structures or partially eroded them.

EOS is remarkably uniform in its internal seismic characteristics, showing multiple bands of strong amplitude reflections, which are traceable throughout the strata (Fig. 2 and Supplementary Figs. 2 and 3), lacking signs like, e.g., moats or sediment waves of large-scale re-deposition in contourite or sheeted drift complexes^30^ or reworking by ocean bottom currents. This points to limited ocean bottom water circulation during this time and (hemi-)pelagic sedimentation.

Questioning strongly increased terrigenous sediments derived from the Antarctic or Australian continents as a major sediment component implies that biogenic sediment comprises a significant portion of the EOS sequences (Fig. 2 and Supplementary Figs. 2 and 3).

Mass balance calculations show an erosional potential of the continental hinterland of Wilkes Land of 2.2 Pt^31^. This expected mass has been calculated based on the identification of relict pre-glacial landscapes onshore such as undulating plateaus below the ice sheet, e.g., covering the Wilkes Subglacial basin, which provide boundary conditions on the amount of material being eroded. The erosional potential is not based on seismic reflection data and indicates further supporting evidence of an additional biogenic source of EOS. The entire post-34 Ma deposition offshore has a mass of 3.48 Pt^19,31^, which exceeds this erosional potential by ~50% and strongly points to an underestimation of biogenic sediments offshore Wilkes Land.

Our computed sedimentation rates and the absence of indicators for major terrigenous sedimentation suggest that EOS experienced additional enhanced biogenic sedimentation. Climate-induced changes to weathering and erosion during the early Oligocene were significant. The extended ice sheet altered established fluvial sedimentation patterns to glacially dominated erosion and transport^8,32^. Furthermore, the increase in continental erosion by physical weathering and associated nutrient availability such as silica increase biodiversity, especially for diatom groups in the early Oligocene^25^. The cored early Oligocene sediments of Site U1356 at the border of EOS exhibit an indicative change in clay mineralogy from chemical (kaolites and smectites) to physical weathering (chlorites and illites)^32^. This changeover in clay mineralogy is corresponding to further indications of stronger mixing in ocean currents and production and/or burial of biogenic matter^17^. The recovered early Oligocene strata is characterized as a silty claystone, which shows signs of increased bioproductivity at the site^17,23^. Comparable high, mainly biogenic sedimentation rates have been observed, e.g., from diatom mats in drill cores in the Neogene Southern Ocean and Equatorial Atlantic^33–35^, which form layers thick enough to be detected in seismic reflection profiles^36^ extending over distances of thousands of kilometers^34,37,38^.

These diatom mats are concentrated beneath oceanic frontal zones, primarily depositing massively on the warm side of the front. The paleoposition of the Oligocene polar front falls within the northern portion of the EOS (Supplementary Fig. 6)^39,40^, supporting additional potential for increased bioproductivity. We propose that the tectonic, oceanographic and climatic configurations in the early Oligocene (34–27 Ma) enabled massive biogenic productivity and associated deposition of the EOS.

### Palaeoceanographic development of the Oligocene AAB

**Fig. 3:**
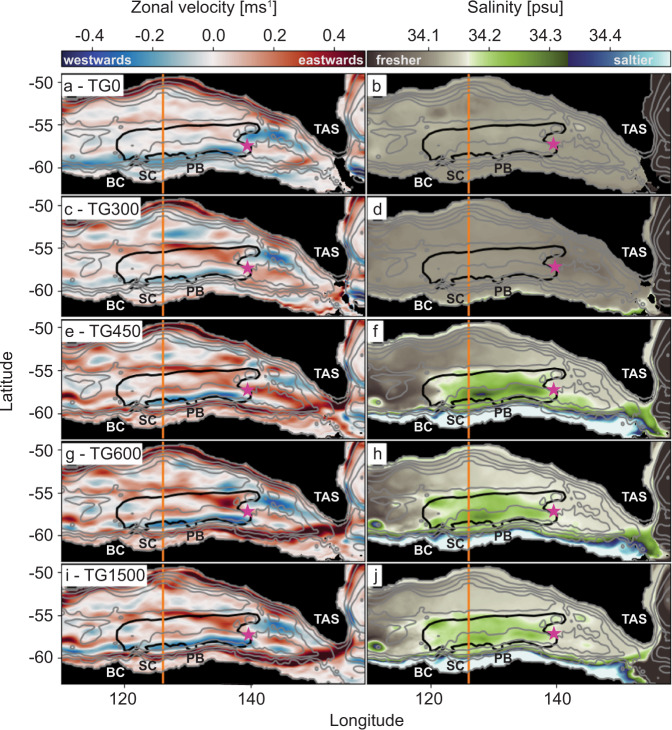
Zonal velocities and salinity in the Oligocene AAB. Mean surface values for **a**, **c**, **e**, **g**, **i** zonal velocities and **b**, **d**, **f**, **h**, **j** salinity within the Australian–Antarctic Basin for different ocean simulations with the Tasmanian Gateway (TG) at **a**, **b** 0 m representing pre-38 Ma, **c**, **d** 300 m representing 38 Ma, **e**, **f** 450 m representing 34 Ma, **g**, **h** 600 m representing ~31 Ma and **i**, **j** 1500 m representing post 27 Ma. Black line is the outline of the Early Oligocene Strata (EOS), and light gray lines are bathymetry contours. The orange line marks the position of the vertical profile (Supplementary Fig. 8) at 126°E corresponding to seismic profile GA228-23 (Fig. 2). The continental features are colored in black. The pink star indicates the location of IODP site U1356. BC Budd Coast, SC Sabrina Coast, PB Porpoise Bay, TAS Tasmania.

To investigate the palaeoceanographic development of the AAB in detail, we use very high-resolution (1/40° grid spacing), submesoscale permitting ocean circulation simulations (Fig. 3 and Supplementary Figs. 7 and 8), with early Oligocene paleobathymetry (Supplementary Fig. 6), paleo-wind stresses, and a deepening Tasmanian Gateway (see Supplementary Information for methods).

**Fig. 4:**
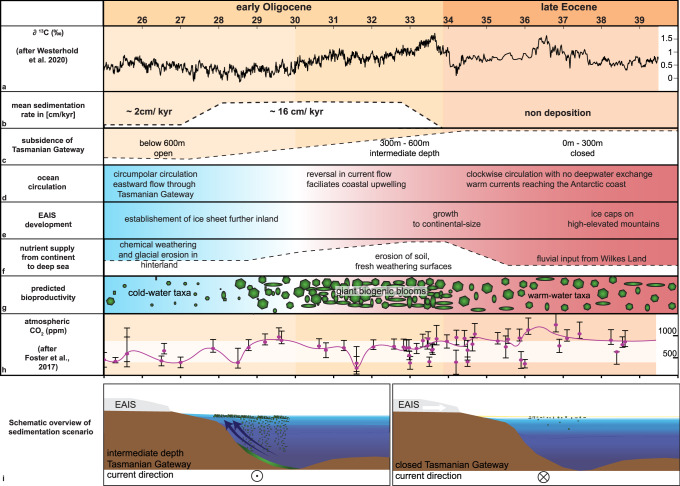
Overview of the development of the Australian–Antarctic Basin between the late Eocene and early Oligocene. **a** ∂^13^C^12^, **b** sedimentation rates (this study), **c** subsidence of the Tasmanian Gateway (Sauermilch et al.^44^), **d** ocean circulation (this study and Villa et al.^51^), **e** East Antarctic Ice Sheet (EAIS) development, **f** nutrient supply from continent (Passchier et al.^32^), **g** predicted bioproductivity (this study), **h** atmospheric CO_2_ (ppm)^12^ with white-shaded area indicating threshold band from 900 to 400 ppm. The error bars of the atmospheric CO_2_ follow the published error bars by Foster et al.^12^. **i** Schematic overview of the sedimentation scenario of a closed and intermediate depth Tasmanian gateway (this study).

When the Tasmanian Gateway is as shallow as 0–300 m, our models show that the AAB is dominated by a strong clockwise gyre (Figs. 3a, c and 4 and Supplementary Fig. 8a, c), warm water (Supplementary Figs. 7a, b and 8a–d), and no upwelling close to the Antarctic coast or lateral advection of saline waters into the basin (Fig. 3a–d). A significant transition occurs with gateway deepening to 450 m. An eastward current appears on the upper Antarctic slope and a westward current over the lower slope (Fig. 3e, f and Supplementary Fig. 8e, f). At the same time, increased spreading of saline water offshore is clearly seen (Fig. 3f). An eastward current favors coastal upwelling in the Southern Hemisphere, as wind-driven Ekman transport offshore forces the colder deep waters to rise. Coastal upwelling systems are known to be baroclinically and barotropically unstable, producing energetic eddies which enhance offshore transport (e.g. ref. 41). The offshore advection seen here is greatest where the slope is very steep, along Budd Coast. This is consistent with heightened instability and eddy production, as also seen near steep escarpments in the Labrador and Nordic Seas^42,43^. The upwelled water is also colder; as it mixes offshore with warmer waters originating in the north, it enhances the distribution of ocean temperatures through the AAB (Supplementary Fig. 7).

This modeled transition is representative of the shift to a glaciated Antarctica at about 34 Ma during which massive amounts of weathered nutrients (e.g., silica and iron) were moved offshore from East Antarctica by glacial expansion. The modeled oceanographic conditions would have transported the nutrients offshore, creating perfect conditions for enhanced bioproductivity including, e.g., massive diatom blooms. A remarkably good match is achieved between our modeled location of saline, presumed nutrient-rich waters and the location of the EOS. Modeled surface temperatures correspond well with proxy data collected at Site U1356^44,45^. An open ocean setting with potentially enhanced bioproductivity is also inferred from early Oligocene samples of drill site U1356 which include abundant specimens of pyritized diatoms as well as a distinct shift in dinocyst assemblage toward a cosmopolitan heterotrophs^17^. Both of these proxies indicate an open ocean setting and are hypothesized to be connected to high primary production resulted from vigorous vertical mixing, which creates seasonal intense blooms^17^.

Our models also demonstrate that the unique oceanographic conditions, necessary for significant offshore transport of nutrient-rich saline water and, therefore, enhanced bioproductivity, weakened with increased deepening of the Tasmanian Gateway from around 30 Ma^40^. Both the eastward and westward currents over the Antarctic slope intensify as the gateway deepens below 600 m (Fig. 3i, j). At the same time, offshore advection of saline water is somewhat reduced (Fig. 3h, j). This suggests the conditions for instability^46^ and offshore eddy transport are greatest when the gateway is of intermediate depth, around 450 m.

### Extreme bioproductivity

Our data and modeling indicate that enhanced seasonal bioproductivity occurred offshore Wilkes Land for 4–7 million years (34 to 30–27 Ma) (Fig. 4). Enhanced bioproductivity initiated by wind-driven upwelling is widely known from bathymetric highs in the Southern Ocean during the mid to late Eocene^36,47–49^ but is typically relatively short-lived^47,50,51^. Here, we propose that the coexistence of warmer proto-Leeuwin waters, the initiation of coastal upwelling, and an associated eddy-driven transport of nutrient-rich waters northward into the center of the AAB, created a regional hub of seasonal productivity lasting 4–7 million years, forming a significant portion of sediment mass of the EOS.

Our suggestion of long periods of high productivity is supported from documented late Eocene opal pulses on oceanic plateaus, which can have durations of up to 4 million years, causing purely biogenic sedimentation rates of up to 4 cm/kyr^48^ and relate to the southward movement and strengthening of the polar frontal system^40,52^. Such extreme events of productivity and rapid burial lead to the deposition of cherts-like sedimentation^36^. The EOS displays multiple bands of high amplitude reflections, which can indicate chert-like biogenic deposition especially in deep sea settings (Fig. 2 and Supplementary Figs. 2 and 3). The viability of prolonged biogenic blooms depends on the supply of a wide array of nutrients as the success of individual plankton families, is closely bound by the accessibility of nutrients and their uptake efficiency^25,53^. Late Eocene plankton blooms on oceanic plateaus are caused by the biological utilization of dissolved silica reaching the surface by enhanced upwelling, and of aeolian input by increased weathering^48^. The high nutrient uptake on bathymetric highs in the Southern Ocean ceases with the Oi-1 glaciation and the final approach of modern δ^30^Si values in the ocean, buffering productivity after the silica pool in surface water masses has been reduced^54^. In the case of the early Oligocene AAB, the nutrient pool recharges constantly through freshly eroded nutrients from the continental hinterland (Figs. 1 and 4) and the coastal upwelling.

The lateral extent and local thickness of the EOS is thus likely related to the onshore topography of East Antarctica and the bathymetry of the continental slope, which likely affects the stability of the eastward and westward jets. Increased run-off, aeolian input and physical weathering, providing crucial nutrients, e.g., iron and silica^35,54^, is enhanced by a dynamic-meltwater rich glaciation on the elevated hinterland^8,31^ areas such as Porpoise Bay (Fig. 1), which is adjacent to the thickest part of the EOS. In comparison, the low-lying areas along the Sabrina Coast^31^ with lower run-off and, therefore, nutrient input show a significantly lower sedimentation rate in the EOS directly offshore (Fig. 1).

### Impact on global CO_2_ drawdown

Given the uniqueness of this regional hub of bioproductivity, it is warranted to carefully explore on its potential impact on the carbon cycle during the early Oligocene and role in the rapid transition from a high CO2 world to moderate CO2 levels and the subsequent climatic stabilization within the middle Oligocene. Timing and seemingly relatively stable conditions align with the second decrease in atmospheric CO_2_ during the early Oligocene (Fig. 1b).

The coupling between bioproductivity, especially siliceous bioproduction, and the biological carbon pump, carbon sequestration, and atmospheric pCO2 is complex^13,15^. Nevertheless, we consider the impact of a significant biogenic source of the EOS on the carbon cycle. Since we do not have samples from within the EOS strata, we are using values from IODP Site U1356 and literature values for this calculation. Further deep ocean scientific drilling will be needed to test the specific composition of the EOS strata and therefore its full potential of carbon drawdown by the EOS strata. Carbon drawdown is an important driver for manifesting colder climatic conditions^13,55^. By assuming a carbon content of 0.5%, the EOS holds 1.067 × 10^15^ kg of organic carbon. This is comparable with a reduction of atmospheric CO2 levels from 900 to 400 ppm, based on estimates of the total atmospheric mass^56^. This reduction in turn manifested the transition toward a permanently glaciated Antarctic continent.

The unique, short-lived positioning of a deepening Tasmanian Gateway and enhanced coastal upwelling, combined with a steady supply of nutrients from vigorous upwelling of deep nutrient-rich waters, the freshly glaciated Wilkes Land margin and strengthened atmospheric circulation, infers that the AAB was a key player in the sequestration of carbon at a tipping point in climate history manifesting colder climates throughout the remaining Cenozoic.

## Methods

### Seismic reflection data

This study uses all currently available multichannel seismic reflection profiles along the Australian sector of the East Antarctic margin, which can be obtained via the SCAR (Scientific Committee of Antarctic Research) Seismic Data Library System (SDLS, http://sdls.ogs.trieste.it/), and along the Southern Australian margin (Geoscience Australia). Most of the data collected in the Australian–Antarctic basin (AAB) were acquired by Australian, Russian and Japanese expeditions since the 1980s. Age control of the key seismic reflectors is achieved by seismic core-log integration with International Ocean Discovery Program (IODP) Expedition 318 Site U1356^23^ for the Antarctic margin. Seismic core-log integration of IODP Site U1356 is performed using the physical properties measured on the core samples and the multi-sensor core logger (MSCL) and transforming them into a synthetic seismic trace under the assumption that the source of the seismic data resembles a Ricker wavelet (Supplementary Fig. 1). Rough weather during the expedition prevented the collection of downhole-logging data^22^. The horizons are identified and traced throughout the seismic network, which allows robust control on the position of the horizons due to the relative high abundance of intersecting profiles (Fig. 1). The horizon WL-U3 is adopted as in Sauermilch et al.^21^ and can be traced throughout the dataset as a strong reflector and seismic unconformity representing a hiatus between 48 and 34 Ma. This reflector pinches out against the oceanic basement deeper in the basin in accordance with magnetic seafloor spreading anomalies^57^ (Fig. 2). The horizon separates bottom-current controlled deposits from the hemipelagic sedimentation above^21^ (Fig. 2 and Supplementary Figs. 1 and 2). At U1356 the unconformity identified as WL-U3 marks the distinct changeover in clay mineral assemblage and therefore weathering conditions from dominantly warm and humid conditions (kaolite) to physical weathering (illite and chlorite)^23,32^. WL-U4 is dated to be 27 Ma, using drill records of IODP Site U1356, and represents the beginning of an increase in sedimentation rate from 30 to 100 m/Myr as well as the resumption of current-driven re-deposition of sediments at the drill site^19,58^ (Fig. 2 and Supplementary Fig. 1). Further to the west offshore the Totten glacier, Donda et al.^28^ identify the onset of channel-levee systems in the upper part of their described Oligocene section, further supporting the onset of an ocean current dominated regime in the late Oligocene. The enclosed seismic unit covers lithological units VI, VII and VIII^23^ at U1356.These units cover early late Oligocene to early Oligocene strata. Unit VI consists of contorted beds of silty claystones with clasts. Units VII and VIII represents interbedded bioturbated claystones with silt-laminated claystones with minimal clast abundance. Especially in the lower Oligocene (lithostratigraphic unit VIII) pyritized diatoms of increased abundance and diversity are preserved implying higher production and/or better preservation than in the underlying Eocene sediments^20^.

To calculate the depth of the seismic reflectors and the volume of the early Oligocene, we use data acquired from sonobuoy and ocean bottom seismometer refraction experiments carried out during the acquisition of the Australian seismic surveys. We follow the method used by Sauermilch et al.^21^ for calculating velocity grids at depth and convert the picked two-way travel time of the horizons to depth. This accounts for the different compaction and sedimentary load of the overlying sediments in different regions of the AAB. For the volume calculation, we used the gmt algorithm grdvolume and included only the area with unusual sedimentation rate (>700 m), resulting in a sediment volume of 0.63 × 10^6^ km^3^. To reveal potential ocean-current-related re-deposition, internal strong reflector bands are traced throughout the EOS, showing level sedimentation in the region of the abyssal plain, pointing to the absence of strong bottom-current activity during the deposition timeframe (Supplementary Fig. 3). For uninterpreted seismic sections of Fig. 2 and Supplementary Fig. 2 refer to Supplementary Figs. 4 and 5.

### Paleobathymetric reconstruction

Reflector horizons represent processes such as the onset of current-related deposition, rather than constant ages. Nevertheless, we postulate that the horizon ages do not vary significantly between the eastern and the western AAB. The cessation and resumption of ocean current-induced reworking of sediments are basin-wide synchronous developments forced by the opening of the Tasmanian Gateway, which form the unconformities. To allow a more appropriate representation of the geometry of the AAB in the ocean model, we reconstructed the paleo-seafloor geometry. To complement the paleobathymetric reconstruction^59^ at the conjugate margin, the top of the Wobbegong formation represents the Eocene/Oligocene Boundary along the conjugate Australian margin^21,60^. Due to abundant exploration wells in several basins of the southern Australian shelf, the age model is well constrained^21,60^. The paleobathymetric calculation is modeled using the BalPal software^61^, which is based on a backstripping technique, including compensation for different crustal types such as continental crust, oceanic crust and stretched continental crust, which is used to reconstruct the Tasmanian Plateau. Furthermore, we correct for global dynamic topography and decompaction of the sediments (for further details on the calculation of the paleobathymetry of the Southern Ocean see Hochmuth et al.^19^). The plate tectonic modeling and the resulting paleolatitudes are computed with the GPlates software using the global reconstructions of Müller et al.^62^ within a paleomagnetic reference frame (Supplementary Fig. 6). This reference frame is best suited for reconstructions including the atmospheric and oceanographic circulation^63,64^ especially for assessing interactions between atmosphere, cryosphere and oceans. This results in a paleolatitude for the Wilkes Land shoreline of ~62°S. The Oligocene Polar front reconstructs after Scher et al.^40^ through the middle of the EOS area (Supplementary Fig. 6). The plate tectonic development through the early Oligocene is mimicked solely by the deepening of the gateways in our ocean models.

### Ocean model

The model runs simulating the response of the ocean across the Eocene-Oligocene transition use the Massachusetts Institute of Technology general circulation model (MITgcm^65^) in an ocean-only regional configuration with no sea-ice. The regional domain extends from 100°E to 165°E in zonal direction and from 65°S to 49°S in meridional direction. The ocean model is run at a submesoscale-permitting resolution of 1/40° with 150 levels in the vertical, ranging from 10 m at the surface to 50 m at the bottom. The model is nested within the 0.25°-resolution ocean model by Sauermilch et al.^44^ and uses its outputs as boundary conditions. Details about the model setup and the implementation of the evolution of the gateways can be found in Sauermilch et al.^44^ (e.g., their Figure  1). The model uses a quadratic drag coefficient of 0.002, a nonlinear equation of state, a seventh order advection scheme and the K-profile parameterization. No parameterization is used for the advection and diffusion due to mesoscale eddies. Partial cells are used for a more accurate representation of bathymetry. The regional configuration uses steady zonal winds (which are the same as in the 0.25° simulations described below), open boundary conditions for temperature, salinity, and meridional/zonal velocities, and a 1°-wide sponge layer at which temperature and salinity are restored with a time scale of 10 days. Five different simulations are run with the only change between simulations being the depth of the Tasmanian Gateway within the parent grid (Supplementary Fig. 6) set at (i) 0 m, (ii) 300 m, (iii) 450 m, (iv) 600 m, and (v) 1500 m, and the values prescribed at the open boundary and in the sponge layer. The depth of the Drake Passage is always constant at 1000 m. The depth of the Drake Passage during this time is unknown. The depth used in this simulation corresponds to regional paleobathymetric reconstructions^66^. In addition, a variation of the depth of the Drake Passage has limited influence on the regional development in the AAB in this model framework. The simulations are run for 8 years, with mean values over the last 2 years shown in the figures.

Boundary conditions for these submesoscale-permitting regional model configurations are taken from a regional model configured for a circumpolar domain and extending from 84°S to 25°S in the meridional direction. This ocean model configuration is run at an eddy-permitting resolution of 0.25° with 50 levels in the vertical, ranging from 10 m at the surface to 368 m at the bottom. Boundary conditions at the surface (sea-surface salinity, sea-surface temperature, zonal/meridional wind stress) and northern boundary (salinity, temperature) are zonal-mean values calculated from a coupled atmosphere-ocean model (GFDL CM2.1) simulating late Eocene conditions with atmospheric CO_2_ concentrations of 800 ppm^12^. Sea-surface salinity and sea-surface temperature are applied via a relaxing boundary condition with a restoring time scale of 10 days. At the northern boundary, a 300-km-wide sponge layer is used to relax zonal-mean values of temperature and salinity with a restoring time scale of 10 days. Each simulation is run for 80 years, with mean fields used for the submesoscale-permitting simulations being a temporal mean over the last 15 years.

Using this nested modeling approach, where boundary conditions from a coarse resolution model are used to force a high-resolution nested model, is currently the only feasible approach to resolve the length scales necessary to accurately simulate the interaction of ocean turbulence, such as submesoscale and mesoscale turbulence, with bathymetry. Large-scale coupled climate models, which cover the global ocean, are computationally too expensive to be run at the required resolution. This nested modeling approach therefore comes with the disadvantage of not being able to simulate large-scale ocean-atmosphere interaction, but instead allows for the study of the role of small-scale turbulence in past climates. Given the crucial role ocean turbulence plays in modern climate, and hence our need to understand the role of ocean turbulence in past climates, highlights the need for more turbulence-resolving regional modeling studies, and should be seen as complementary to coupled global climate models, with both approaches having their advantages and disadvantages.

In this simulation, the forcing of the ocean by the atmosphere is fixed, and hence there can be no feedbacks between the ocean and the atmosphere. Since the regional model configuration used in this work uses boundary conditions from an eddy-permitting simulation of the full circumpolar extent of the Southern Ocean, the boundary conditions take changes in the circumpolar dynamics, such as for example the transport of the Antarctic Circumpolar Current into account. Since the results presented here depend on the large-scale reorganization of ocean currents from gyres to a circumpolar current, this approach should be adequate to understand the ocean dynamics, associated with the interaction of jets with topography, which are crucial as prerequisite to support the biogenic blooms discussed here. Nevertheless, understanding the possible role of atmosphere-ocean feedbacks for these dynamics would require a submesoscale-permitting coupled model which is clearly beyond the scope of this paper.

### Calculation of carbon content of the EOS

Since no drill samples of the EOS have been recovered outside of its marginal area at IODP Site U1356, we rely on literature values for the calculation of the carbon content of the EOS. The carbon content within sediments depends mainly on two factors, the productivity in the water column and the burial rate at the seafloor. The global comparison of sediment samples reveals a strong correlation between the sedimentation rate and the carbon content of the sample. For medium sedimentation rates between 2 and 13 cm/kyr carbon content in sediment can be expected to range from 0.1 to 2%^67,68^. Even though our mean sedimentation rates are slightly higher (16 cm/kyr), we conservatively calculate the carbon content as 0.5% carbon within the sediments as a general approximation, resulting in total carbon of 1.067 × 10^15^ kg. To reduce the atmospheric carbon content by 1 ppm, 2.1 × 10^12^ kg of carbon need to be sequestered^55^.

## Supplementary information

Supplementary Information

Inventory of Supplement

## Data Availability

The paleobathymetric data used in this study have been deposited in the Pangaea database under accession code 10.1594/PANGAEA.918663. The ocean model outputs generated in this study has been made available on the Zenodo database under the accession code 10.5281/zenodo.7222744. Reflection seismic data used in this study are available at the SCAR (Scientific Committee of Antarctic Research) Seismic Data Library System (SDLS, http://sdls.ogs.trieste.it/).

## Code availability

The code used in this study can be obtained from the corresponding author.
