# Supplementary material for: Southern Ocean biogenic blooms freezing-in Oligocene colder climates
Source: Nat Commun. 2022 Nov 9;13:6785. doi: 10.1038/s41467-022-34623-9 (PMC9646741; doi:10.1038/s41467-022-34623-9)
Supplement: Supplementary file 1 — Supplementary Information [file 41467_2022_34623_MOESM1_ESM.pdf]

Supplementary Information to

Southern Ocean biogenic blooms freezing-in Oligocene colder climates

Katharina Hochmuth[1,2,3*], Joanne M. Whittaker[2,3], Isabel Sauermilch[4], Andreas Klocker[5,6], Karsten Gohl[7], Joseph H. LaCasce[5]

[1] School of Geography, Geology and the Environment, University of Leicester, UK
[2]Institute for Marine and Antarctic Studies, University of Tasmania, Hobart, Australia
[3] Australian Center for Excellence in Antarctic Sciences, University of Tasmania, Hobart, Australia
[4] Department of Earth Sciences, Faculty of Geosciences, Utrecht University, Utrecht, The Netherlands
[5]Department of Geosciences, University of Oslo, Oslo, Norway
[6]now at NORCE Norwegian Research Centre, Bjerknes Centre for Climate Research, Bergen, Norway
[7] Alfred Wegener Institute Helmholtz-Centre for Polar and Marine Research, Bremerhaven, Germany

[*]corresponding author: Katharina.Hochmuth@utas.edu.au

Supplementary Figures:

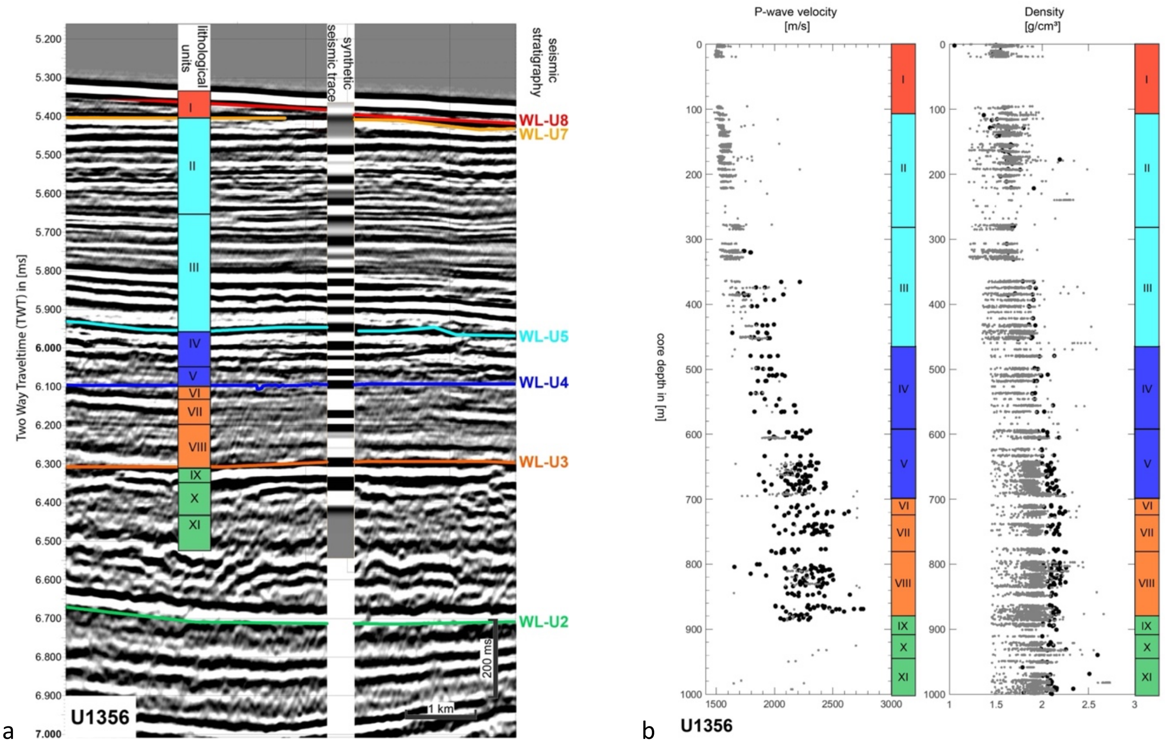

Supplementary Figure 1: Core-log seismic integration of IODP Site U1356

a) Core-log seismic integration: Synthetic seismogram of IODP Site U1356 correlated into seismic line GA-228-29. Lithostratigraphic units after[23].

b) IODP Site U1356 sediment-physical properties: P-wave and density measurements on core samples (black) and along core (grey) of U1365 and corresponding lithostratigraphic units[23]. The Early Oligocene strata (EOS), discussed in this study corresponds to lithostratigraphic unit VIII.

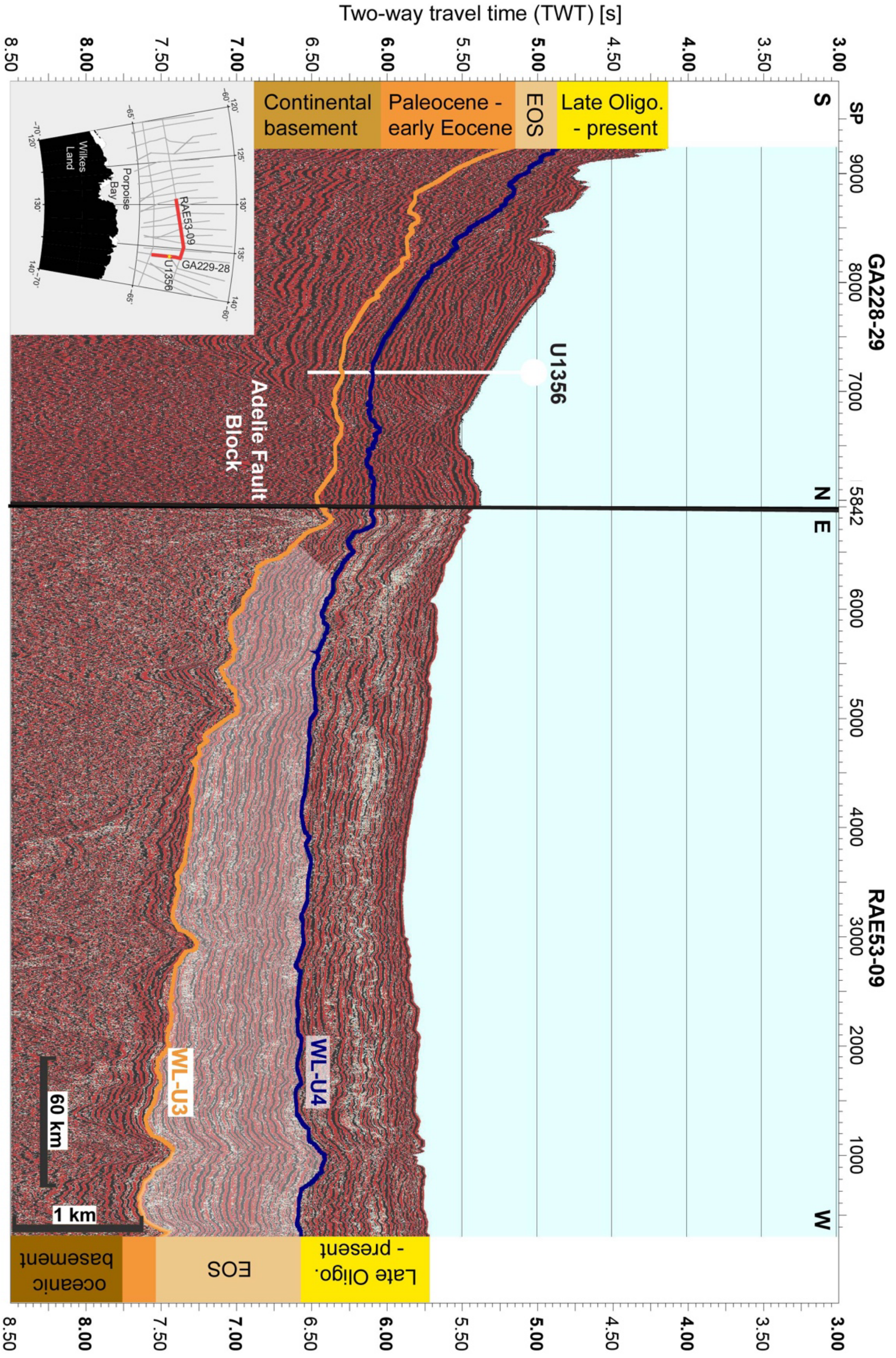

Supplementary Figure 2:
Seismic transect between IODP Site U1356 and EOS strata: Combined seismic profiles GA228-29 and RAE53-09 showing the connection of IODP Site U1356 across the Adelie fault block and towards the center of the EOS (shaded area). Note: the different amplitude intensity of the seismic section is a result of the different equipment and acquisition parameters used during the two separate expeditions.

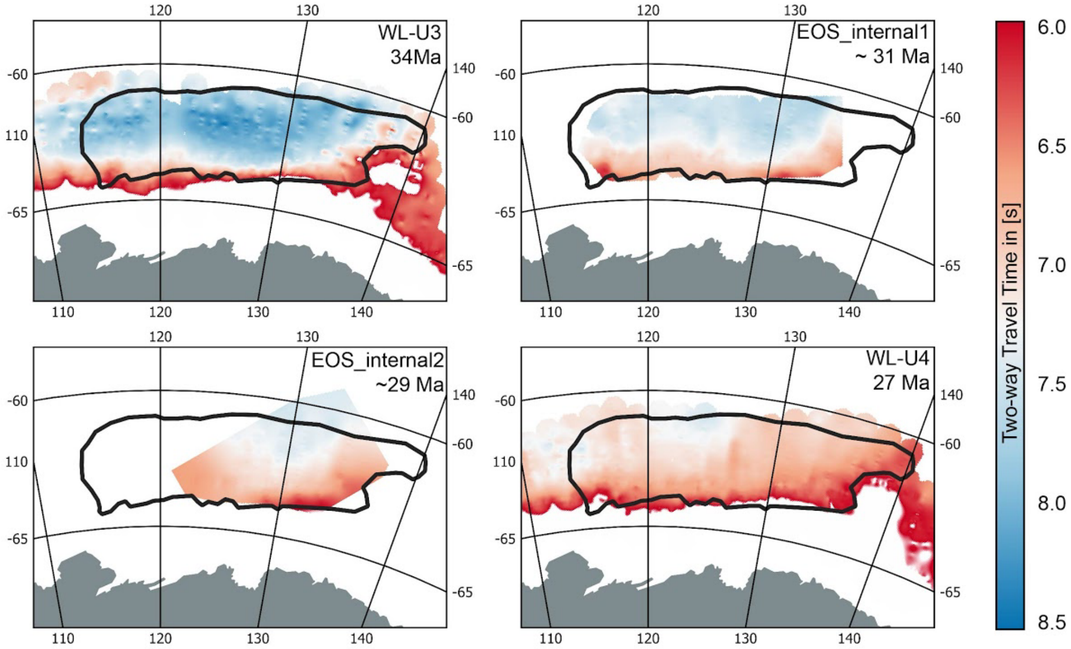

Supplementary Figure 3:

Maps of internal reflectors within EOS strata: Seismic reflector grids of the bounding reflectors WL-U3 (34 Ma) and WL-U4 (27 Ma), as well as strong reflector bands within the strata (EOS_internal1 and EOS_internal2); assuming a constant sedimentation rate between WAL-U3 and WL-U4, the age of the internal reflectors are ~31 Ma and ~29 Ma, respectively. The younger EOS_internal2 can only be traced in the thickest part of the formation; black line indicates the outline of the EOS, all horizons are remarkably flat and do not indicate drift building features such as moats.

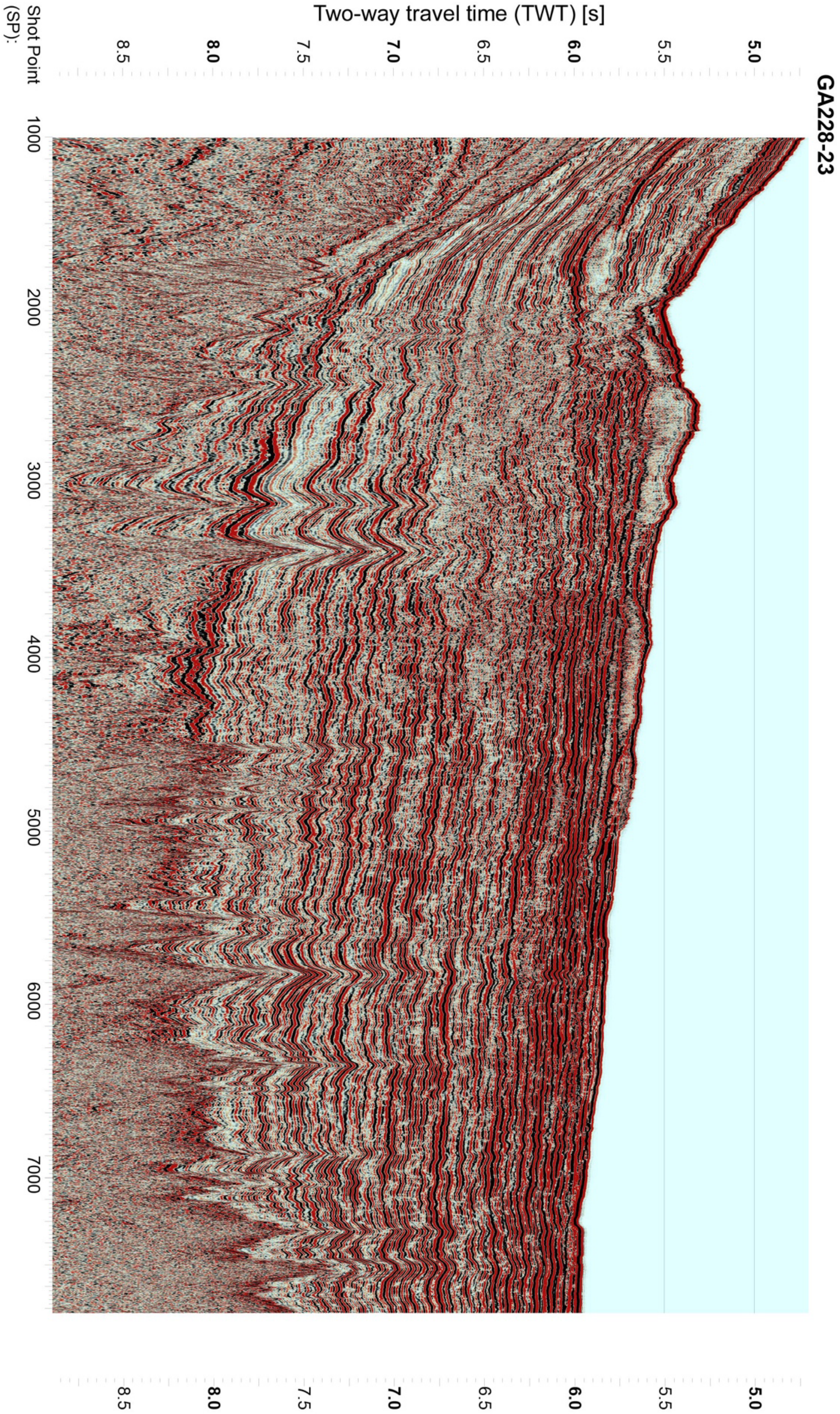

Supplementary Figure 4:
Uninterpreted version of Figure 2: Uninterpreted seismic section GA-228-23; The interpreted version and location are shown as Fig. 2 in the main text; Seafloor magnetic anomaly identifications[56] span ages from Early Cretaceous (C34n) to the early Eocene (C20n).

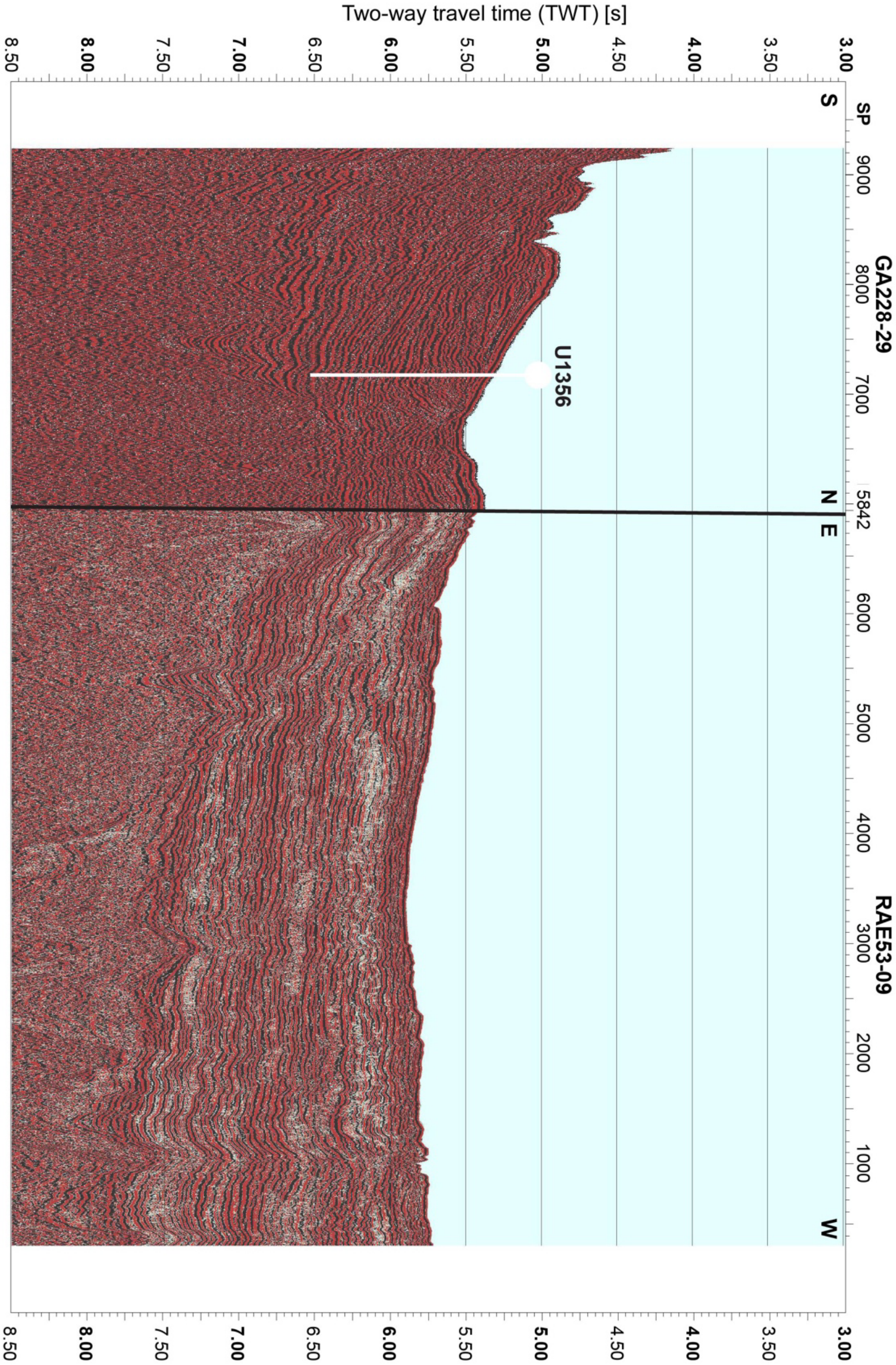

Supplementary Figure 5:
Uninterpreted version of Supplementary Figure 2: Uninterpreted seismic sections GA-228-29 and RAE53-09. White line indicates position and penetration depth of IODP Site U1356. The interpreted section and the profile location are shown in Supplementary Figure 2. Note: the different amplitude intensity of the seismic section is a result of the different equipment and acquisition parameters used during the two separate expeditions.

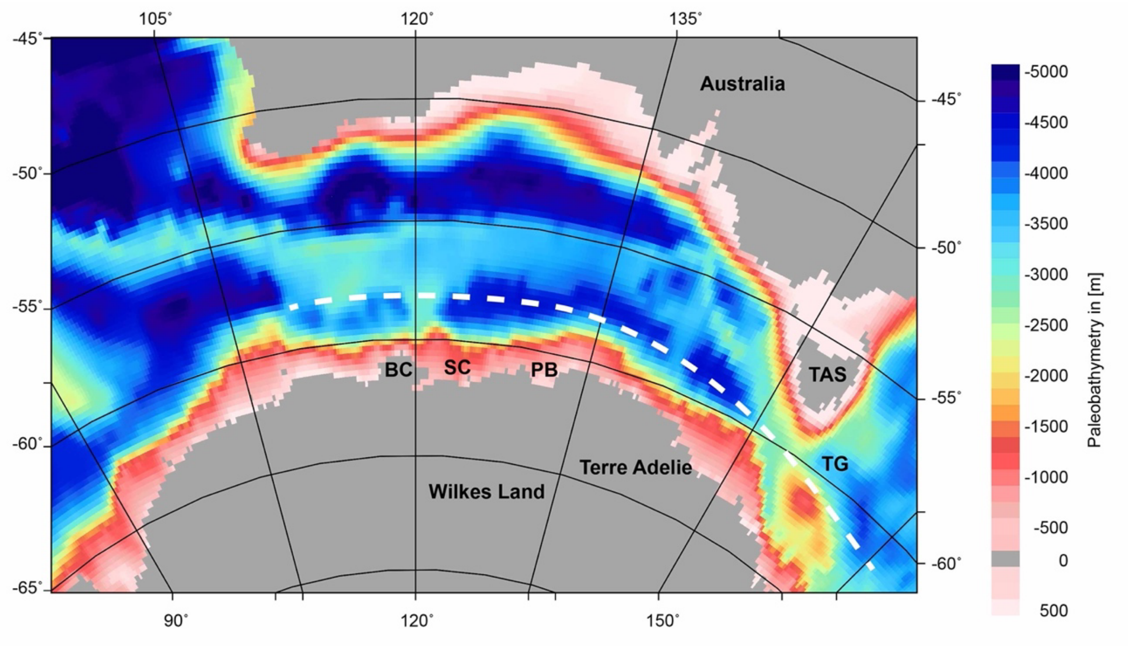

Supplementary Figure 6:
Early Oligocene bathymetry of the Australian Antarctic Basin: Paleobathymetry[19] used in the ocean model for the Australian Antarctic Basin; grey areas on the map indicate continental areas, which have been set to 0m elevation in the ocean models (Fig. 3 & Supp. Figure 7), depth of the Tasmanian Gateway and Drake Passage have been adjusted in our ocean models to reflect the deepening of the gateway through the early Oligocene comparable to Sauermilch et al.[44](see their figure 1). The white dashed line indicates the reconstructed location of the polar front during the early Oligocene[40] BC Budd Coast, SC Sabrina Coast, PB Porpoise Bay, TAS Tasmania, TG Tasmanian Gateway

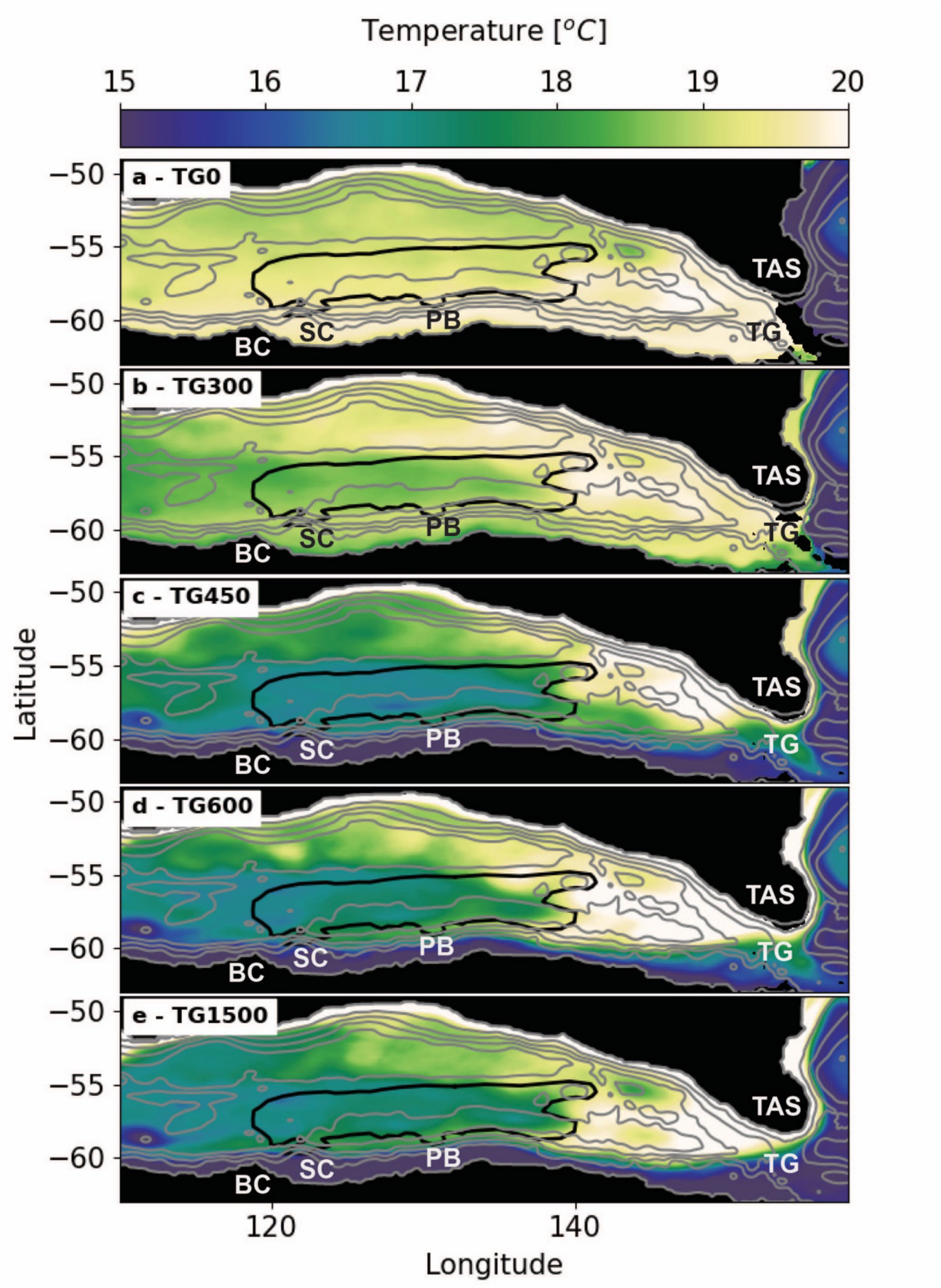

Supplementary Figure 7:

Early Oligocene surface temperatures: Surface temperatures within the Australian-Antarctic Basin for different ocean simulations with the Tasmanian Gateway (TG) at (a) 0 m representing pre-38 Ma, (b) 300 m representing 38 Ma, (c) 450 m representing 34 Ma, (d) 600 m representing ~31 Ma and (e) 1500 m representing post-27 Ma. Black line is the outline of the Early Oligocene Strata (EOS), and grey lines are bathymetry contours. BC Budd Coast; SC Sabrina Coast; PB Porpoise Bay; TAS Tasmania.

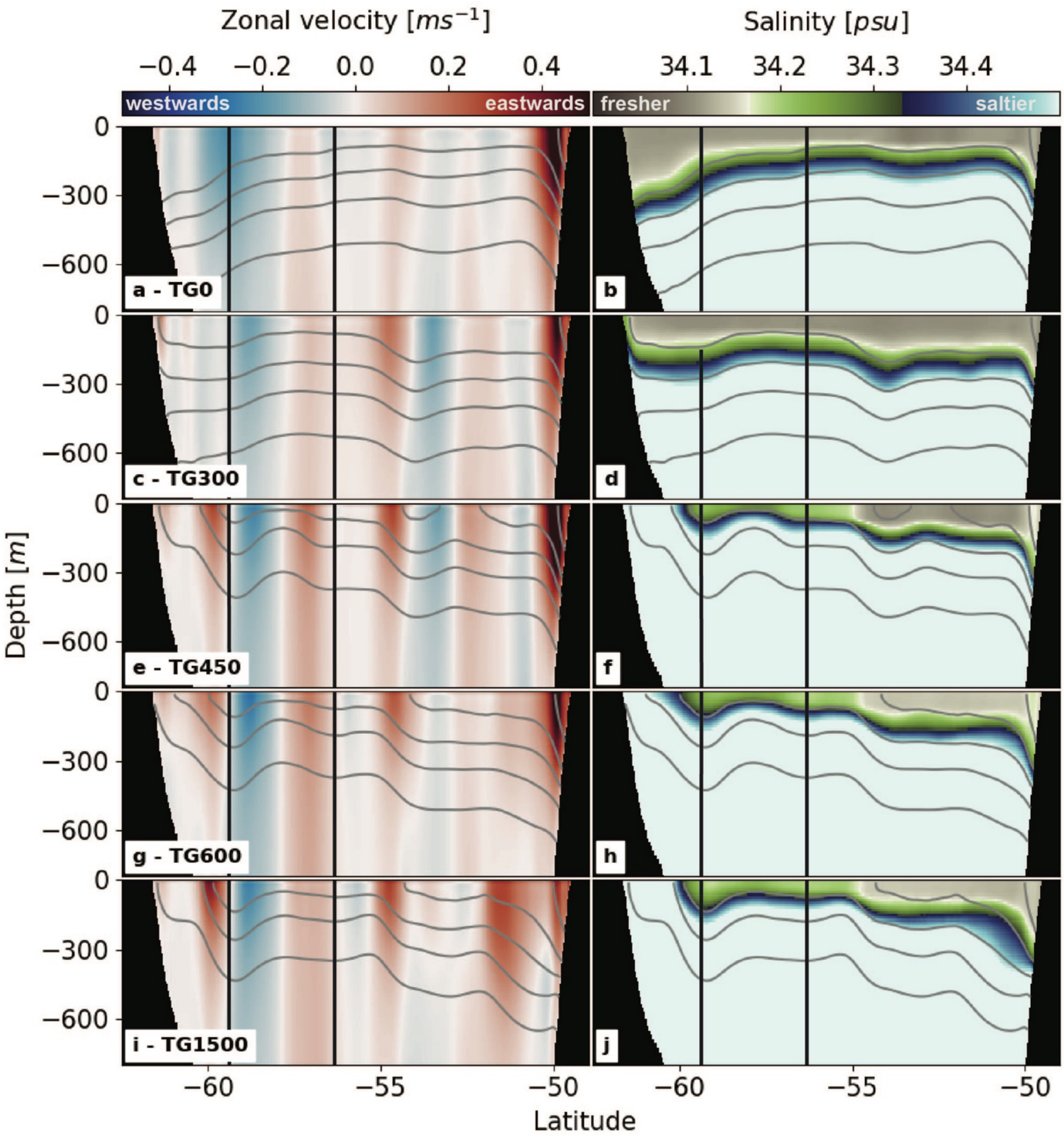

Supplementary Figure 8:

Ocean model transect at 126˚ E: Vertical section of zonal velocities and salinity at 126˚ E/GA228-23 (Fig. 2) (see Fig. 3 for exact location). Panels representing different ocean simulations with the Tasmanian Gateway (TG) at (a, b) 0 m representing pre-38 Ma, (c,d) 300 m representing 38 Ma, (e,f) 450 m representing 34 Ma, (g,h) 600 m representing ~31 Ma and (I,j) 1500 m representing post-27 Ma. Grey lines are surfaces of constant density. Black lines indicate the position of the southern and northern boundary of the EOS strata.
