# Supplementary material for: Southern Ocean biogenic blooms freezing-in Oligocene colder climates
Source: Nat Commun. 2022 Nov 9;13:6785. doi: 10.1038/s41467-022-34623-9 (PMC9646741; doi:10.1038/s41467-022-34623-9)
Supplement: Supplementary file 2 — Inventory of Supplement [file 41467_2022_34623_MOESM2_ESM.docx]

**Inventory of Supplementary Infromation**

**Southern Ocean biogenic blooms freezing-in Oligocene colder climates**

Katharina Hochmuth^1,2,3*^, Joanne M. Whittaker^2,3^, Isabel Sauermilch^4^, Andreas Klocker^5,6^, Karsten Gohl^7^, Joseph H. LaCasce^5^

Supplementary Figures:

Supplementary Figure 1: Core-log seismic integration IODP Site U1356

Supplementary Figure 2: Seismic transect between IODP Site 1356 and EOS strata

Supplementary Figure 3: Maps of internal reflectors within EOS strata

Supplementary Figure 4: Uninterpreted version of Figure 2

Supplementary Figure 5: Uninterpreted version of Supplementary Figure 2

Supplementary Figure 6: Early Oligocene bathymetry of the Australian Antarctic Basin

Supplementary Figure 7: Early Oligocene surface temperatures

Supplementary Figure 8: Ocean model transect at 126˚ E
